# Socioeconomic and lifestyle factors predict the association between sleep health and depression

**DOI:** 10.64898/2026.06.26.26356679

**Published:** 2026-06-29

**Authors:** Wen Liu, Vincent Küppers, Hanwen Bi, Mostafa Mahdipour, Jianxiao Wu, Fateme Samea, Felix Hoffstaedter, Kathrin Wolf, Charlotte von Gall, Agustin Ibanez, Simon B. Eickhoff, Sarah Genon, Somayeh Maleki Balajoo, Masoud Tahmasian

**Affiliations:** 1. Institute of Neuroscience and Medicine, Brain and Behaviour (INM 7), Research Center Jülich, Jülich, Germany; 2. Institute of Systems Neuroscience, Medical Faculty and University Hospital Düsseldorf, Heinrich Heine University Düsseldorf, Düsseldorf, Germany; 3. Department of Nuclear Medicine, Faculty of Medicine and University Hospital Cologne, University of Cologne, Cologne, Germany; 4. Clinic and Polyclinic for Child and Adolescent Psychiatry, Psychotherapy, and Psychosomatics, Medical Faculty, Leipzig University, Leipzig, Germany; 5. Institute of Epidemiology, Helmholtz Zentrum München GmbH, German Research Center for Environmental Health, Neuherberg, Germany; 6. Institute of Anatomy II, Medical Faculty, Heinrich Heine University, Düsseldorf, Germany; 7. Latin America Brain Health Institute (BrainLat), Universidad Adolfo Ibanez, Santiago, Chile; 8. Global Brain Health Institute (GBHI), Trinity College Dublin, Dublin, Ireland; 9. Department of Biophysics, School of Medicine, Istanbul Medipol University, Istanbul, Türkiye; 10. Barcelonaβeta Brain Research Center (BBRC), Pasqual Maragall Foundation, Barcelona, Spain; 11. Cognitive Neuroscience Center (CNC), Universidad de San Andrés, Buenos Aires, Argentina; 12. GIGA-CRC-Human Imaging, University of Liege, Liège, Belgium

## Abstract

**Objective:**

Sleep health and depression are interconnected multidimensional constructs, yet their shared determinants remain obscure. Understanding the role of socioeconomic/lifestyle factors in predicting sleep-related depression (SRD) is critical for preventive strategies. This study aimed to identify the key socioeconomic/lifestyle predictors of SRD in the general population and patients with clinical depression.

**Methods:**

To characterize SRD, we performed regularized canonical correlation analysis between sleep and depression to identify latent phenotypes of SRD in a general population subsample (GP1; n = 87,405) from the UK Biobank. Subsequently, machine-learning predictive models were developed in GP1 to predict SRD using socioeconomic/lifestyle factors. The best-performing predictive model was subsequently validated in GP2 at both baseline and follow-up (GP2; n = 5,187), and in clinical depression (n = 7,454) to assess its generalizability. Complementary analyses were conducted to assess other latent phenotypes (i.e., depression-related sleep, non-SRD, non-depression-related sleep, overall sleep health, and overall depression).

**Results:**

A robust multivariate association was identified between sleep and depression in GP1 (canonical r = 0.42, P_FDR_ < 0.001). Socioeconomic/lifestyle factors moderately predicted SRD (r = 0.25; 95% CI: [0.24, 0.25]; R^2^ = 0.06; 95% CI: [0.06, 0.06]; rMSE = 1.08; 95% CI: [1.08, 1.09]). The top predictors were less frequency of confiding in others, more sedentary television viewing, less vigorous physical activity, and passive smoking exposure. Out-of-sample validation of the predictive model showed similar patterns in GP2 at baseline, at follow-up, and in clinical depression subsamples. Similarly, less frequency of confiding in others and greater sedentary television viewing were the main predictors of other depression-related profiles, whereas more alcohol consumption frequency, less walking frequency, and less time spent outdoors in winter predicted poor sleep-related profiles.

**Conclusions:**

Our generalizable predictive model identifies critical modifiable predictors of the association between sleep health and depression that could serve as potential targets for personalized interventions.

## Introduction

Depression is the leading cause of disability worldwide, ranging from subclinical depressive symptoms in the general population to patients with major depressive disorder (MDD). Depression is inherently multidimensional, characterized by substantial heterogeneity across emotional, cognitive, social, biological, somatic, and sleep domains.^1,2^ Poor Sleep health (SH) represents pervasive features and a core domain of depression.^1,3,4^ Approximately 78% of patients with MDD report insomnia symptoms.^5^ SH is a multifaceted construct encompassing sleep duration, continuity, and quality, which support optimal daytime functioning, mental health, and well-being.^6,7^ Individuals with sleep disturbances and insomnia have a greater risk for the onset, recurrence, and exacerbation of MDD.^8–13^ Meanwhile, sleep disturbances are also considered a diagnostic criterion for MDD^2^, reflecting their bidirectional relationship. Beyond symptom-level associations, converging genetic and neuroimaging evidence further underscores a tight coupling between poor SH and MDD,^14–17^ supporting a robust, multidimensional relationship and a shared mechanism underlying the sleep-depression nexus. However, prior studies predominantly relied on group-level comparisons or univariate correlations examining individual sleep characteristics (e.g., insomnia) and overall depression (total scores) in isolation, either in the general population or in patients with MDD.^18–20^ These approaches overlook inter-individual variability and fail to capture the complex, multifaceted nature of their associations, or their generalizability across populations. A multivariate framework is therefore needed to disentangle these overlapping constructs and identify their shared multidimensional structure. Thus, we introduce *sleep-related depression (SRD)* as a novel construct capturing the specific dimension of depressive symptoms that strongly covaries with SH characteristics at the individual level.

The modifiable factors contributing to their shared variation remain poorly understood, particularly at the individual level across both the general population and MDD samples. Socioeconomic and lifestyle factors are modifiable exposures that influence the severity of both depression^21,22^ and sleep problems^23,24^. Poverty, social isolation, and unhealthy lifestyle, including physical inactivity, malnourishing diet, smoking, and high alcohol consumption, are associated with an increased risk of depressive symptoms and onset of MDD,^25–27^ whereas healthy lifestyle and social support are protective factors.^27,28^ Despite well-documented group-level evidence, existing works have examined socioeconomic and lifestyle factors in relation to either SH or depression. Consequently, it remains unclear whether and how these factors shape the shared variance between SH and depression, e.g., by predicting SRD.

This study leveraged large-scale data and multivariate association analyses to derive individual-level SRD scores, representing canonical variates of depression that maximized their correlation with corresponding SH canonical variates — thereby translating shared SH–depression variance into an individual-level metric beyond conventional group-level measures. Machine learning (ML) models were then employed to predict SRD scores from socioeconomic/lifestyle factors and quantify their relative contributions to the shared SH-depression phenotype in the general population. Model generalizability was evaluated in independent general population subsamples (GP), both cross-sectionally and longitudinally, and in a clinical depression subsample (CD). Using complementary analyses, we examined the differential contributions of these factors across additional latent phenotype profiles, including depression-related sleep (DRS), non-sleep-related depression (NSRD), non-depression-related sleep (NDRS), overall SH, and overall depression. This systematic approach was adopted to disentangle the predictive role of socioeconomic/lifestyle factors on the shared and distinct latent dimensions underlying the SH and depression.

## Methods

### Participants and data handling

Data were obtained from the UK Biobank (UKB, ethical approval in eMethods 1). To calculate SRD scores, we analyzed four domains in both GP and CD: *i)* seven SH components; *ii)* depressive symptoms; *iii)* socioeconomic variables; and *iv)* lifestyle factors (eMethods 1, eTable 1). Variable selection was based on the availability of UKB data.

**Figure 1:**
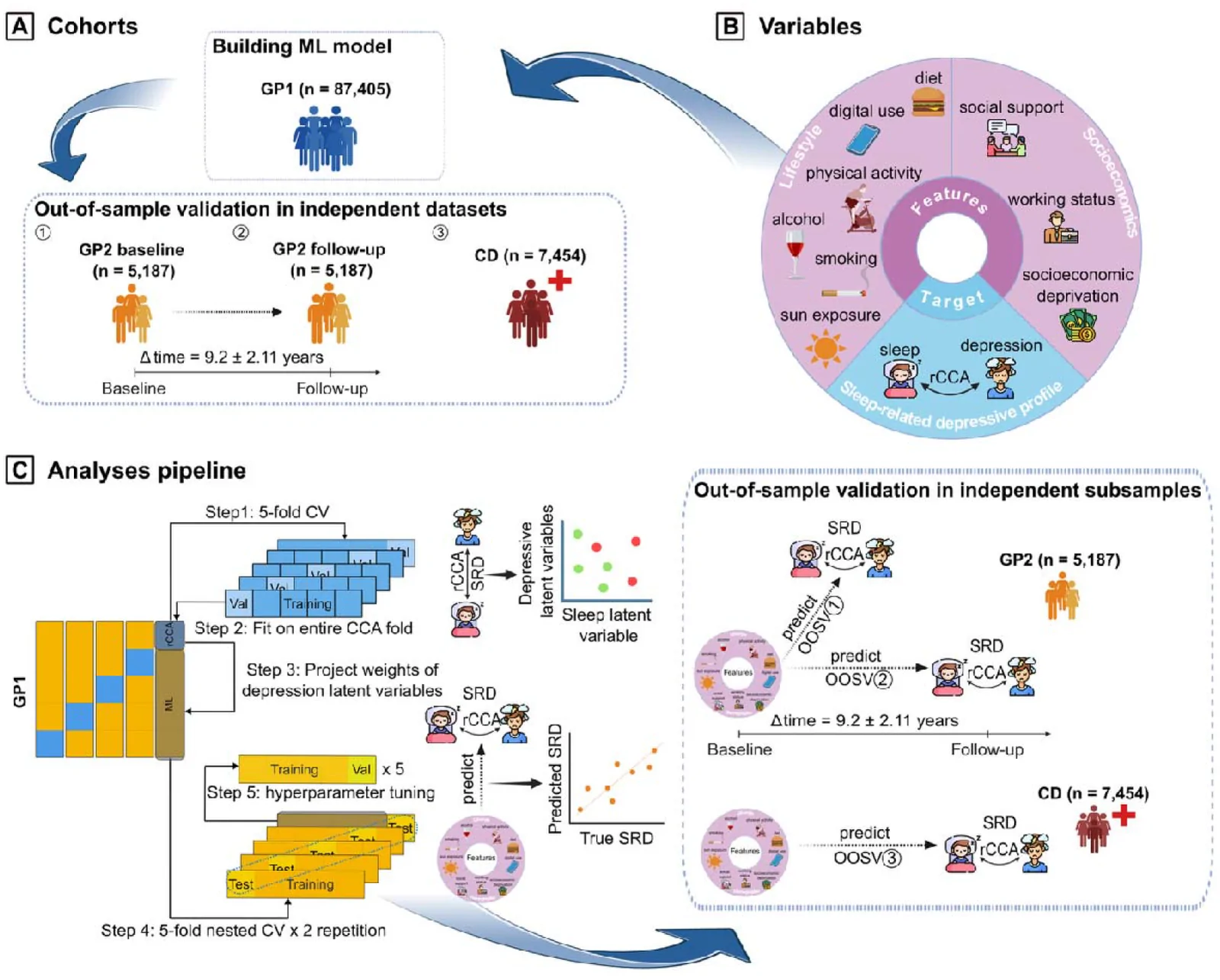
Overview of design and analyses. **A.** The UK Biobank cohort was divided into four independent subsamples. The largest one is for model training and testing (GP1), and three independent subsamples are used for out-of-sample validation (GP2 at baseline and at follow-up, as well as CD). **B.** Overview of variables as socioeconomic and lifestyle (input features) and sleep-related depression (SRD) profile (target) in the predictive model. **C.** rCCA identified the strongest multivariate association between SH and depression to obtain the individual-level SRD scores. Subsequently, linear and non-linear ML models were trained to predict SRD scores based on lifestyle and socioeconomic factors. The rCCA and predictive procedures were implemented in GP1 within an integrated nested cross-validation framework, with one fold for rCCA and the other four folds for prediction to avoid data leakage. Out-of-sample validation was performed on three independent samples to assess the generalizability of the predictive model trained and tested in GP1. GP: general population; CD: clinical depression; OOSV: out-of-sample validation; CV: crossvalidation; rCCA: regularized canonical correlation analysis; ML: machine learning.

Participants were included if they had complete data across all domains and met our selection criteria (eTable 2–3, eMethods 2). Eligible participants were divided into four independent subsamples (Figure 1A). The primary subsample was GP1 (n = 87,405) with cross-sectional data, used to derive the SRD scores, ML predictive models, and complementary analyses. Generalizability of predictive models was assessed in independent subsamples using both baseline (GP2 baseline; n = 5,187) and follow-up (GP2 follow-up; mean follow-up 9.2 years; n = 5,187) assessments to evaluate the long-term roles of baseline socioeconomic/lifestyle factors in predicting follow-up SRD scores. We also assessed multivariate associations and the generalizability of predictive models in CD (n = 7,454). Participants in CD either had a documented ICD-10 diagnosis of either current depressive episode (F32) or recurrent depressive disorder (F33) or were taking antidepressant medication (eMethods 2) upon assessment.

### Statistical Analyses

#### Analysis framework

To maximize data utilization, obtain unbiased individual-level SRD estimates, and prevent data leakage, we novelly combined regularized canonical correlation analysis (rCCA) and predictive modeling within a nested cross-validation framework^29^ (Figure 1C). The GP1 was randomly split into five outer folds. In each iteration, one outer fold (CCA fold, 20%) was used to build the rCCA model, while the remaining four folds (ML fold, 80%) were used for predictive models (Figure 1C). Within each CCA and ML fold, a nested cross-validation scheme was conducted.^30^ To prevent data leakage, age and sex were regressed out using only training data, and the resulting regression parameters were applied to the validation and test sets for both rCCA and predictive models.^31^ (eMethods 3)

#### Regularized Canonical Correlation Analysis

RCCA is a multivariate approach that quantifies the maximum shared variance between at least two sets of variables while reducing overfitting through regularization, as previously applied.^1,32–34^ We applied rCCA in GP1 and CD to calculate SRD scores, reflecting the multivariate association between SH and depression characteristics after controlling for age and sex as covariates. The first canonical variate of depression, capturing the dimension most strongly associated with SH, was used to represent individual-level SRD scores. Canonical loadings were calculated as the associations between the original variable and its corresponding canonical variate. Redundancy was calculated as the mean variance of one set explained by the canonical variates of the other set.^35^ To minimize overfitting and enhance robustness and generalizability, a regularized version (rCCA) was implemented within a ML framework,^1,32,36^ with a hyperparameter optimization procedure described in eMethods 4. Statistical significance for rCCA was assessed using a false discovery rate (FDR) correction with a threshold of *P*_*FDR*_ < 0.05 (eMethods 5).

#### Predictive analyses

To evaluate whether socioeconomic/lifestyle features can predict SRD scores, several linear and non-linear ML models, including ridge regression,^37^ LASSO regression,^38^ elastic net,^39^ random forest,^40^ extreme gradient boosting (XGBoost),^41^ linear support vector regression (SVR),^42^ and SVR with radial basis function kernel^43^ were trained in GP1 (Figure 1B, eMethods). Multicollinearity among features was controlled using the variance inflation factor (VIF), with values > 5 indicating potentially problematic multicollinearity.^44^ The best-performing model was calibrated (eMethods 6), and compared with a demographic model including age, sex, and years and education as predictors to assess whether the predictive model captured target-relevant information beyond demographic characteristics alone. The best-performing model with and without controlling confounds were compared to evaluate the potential risk from linear confound adjustment into non-linear models.^45^

Feature contributions were quantified using SHapley Additive exPlanations (SHAP) values and examined across age and sex subgroups to assess potential heterogeneity in feature contributions. Age- and sex-stratified analyses were conducted within the best-performing predictive model using a threshold of *P*_*FDR*_ < 0.05 for statistical significance (eMethods 7). The best-performing predictive model trained in GP1 was then validated in three independent subsamples (GP2 baseline, GP2 follow-up, and CD) to evaluate generalizability across populations using out-of-sample validation (eMethods 8).

#### Complementary analyses

We examined whether and how socioeconomic/lifestyle factors contributed differently to predicting other profiles of distinct latent phenotypes, including DRS, NSRD, NDRS, overall depression, and overall SH (eMethods 9). The same socioeconomic/lifestyle features were applied to GP1 using XGBoost to predict each latent profile. To derive latent dimensions comparable to SRD, principal component analysis was performed on each profile, and the first component, representing the largest proportion of variance, was selected as the representative dimension.

We also conducted sensitivity predictive analyses in CD by excluding participants with 1) recurrent depression (n = 91) and 2) both recurrent depression and antidepressant use (n = 5,301, eTable 4) to control for their impact.

## Results

### Demographics and phenotypic characteristics

The primary rCCA, ML analyses, and complementary analyses were conducted in GP1. Out-of-sample validation was performed in baseline and follow-up GP2 and CD (demographic and feature characteristics in eTable 5).

### Multivariate associations between SH and depression

**Figure 2:**
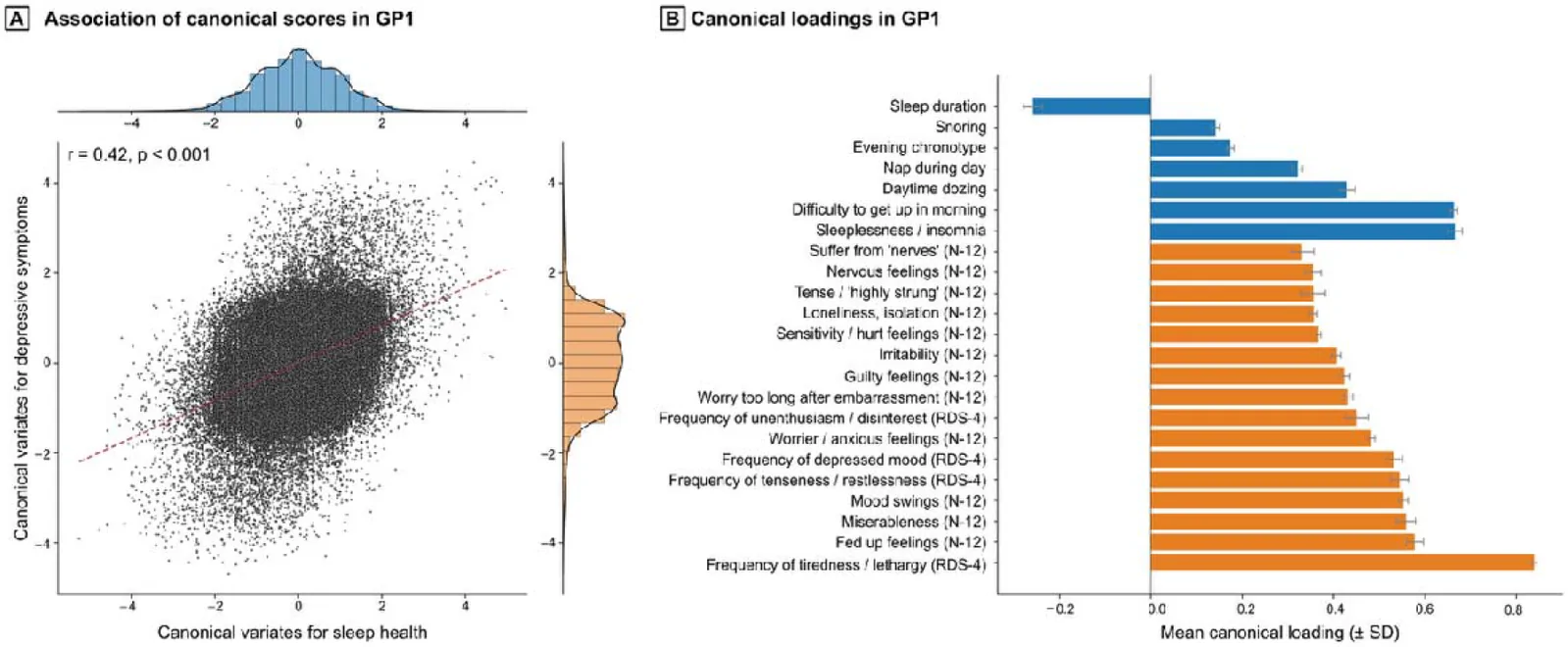
First significant latent dimension and loadings for sleep health (SH) and depression in the general population 1 (GP1) to calculate the sleep-related depression (SRD) profile. **A.** Multivariate associations between canonical variates for SH and canonical variates of depression in GP1. Each point represents an individual-level SRD score, with scores averaged across the test sets. Model performance is illustrated using the correlation coefficient (r). The blue and orange bar plots show the distributions of latent scores for SH and depression, respectively. **B.** Mean loadings across five outer CCA folds. Error bars indicate one standard deviation. Greater disturbances in SH loadings were associated with higher depression loadings. The blue bars represent SH, and the orange ones reflect depression. N-12: neuroticism-12; RDS-4: recent depressive symptoms, indicating depressive symptoms in the past two weeks.

The first canonical dimension captured the strongest shared variance (17.6%) between SH and depression. Our rCCA results in GP1 showed a positive association between the corresponding canonical variates (canonical correlation r_mean_ = 0.42 [SD: 0.002], *P*_*FDR*_ < 0.001; Figure 2A). The canonical variates of depression from this dimension were therefore used to measure SRD scores. These individual-level SRD scores were used as the target for predictive models.

All SH components were positively associated with depression, except for sleep duration, for which shorter duration was associated with more severe depression. Among all components in SH, insomnia and difficulty getting up in the morning contributed most strongly to the SH variate, whereas frequency of tiredness or lethargy over the past two weeks showed the highest loading among depression measures. Collectively, an individual with a higher SRD score had more sleep disturbances and greater depression severity (Figure 2B). The proportion of SH variance explained by SRD in GP1 was in eTable 6.

### Predictability of SRD from lifestyle/socioeconomic factors in GP1

**Figure 3:**
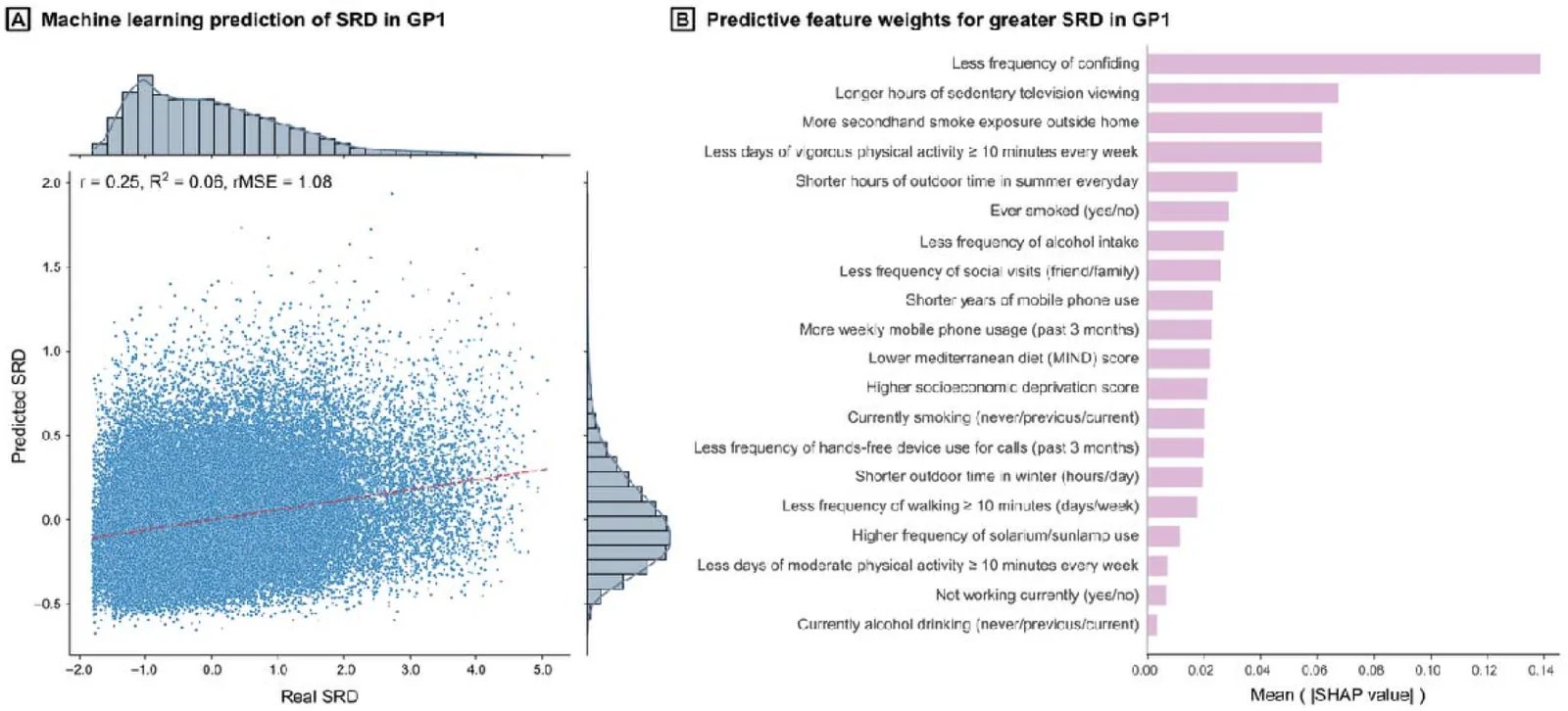
Predictive model performance in general population 1 (GP1). **A.** Each point represents an individual; bar plots represent the distribution of real and predicted SRD scores. Model performance is illustrated using the correlation coefficient (r), coefficient of determination (R^2^), and root mean square error (rMSE). **B.** Weights for each sociodemographic and lifestyle feature in predicting higher SRD in GP1. The length of each bar represents the mean absolute SHAP values across the split test sets, reflecting the importance of each lifestyle and sociodemographic factor in predicting higher SRD. GP: general population subsample; SRD: sleep-related depression; SHAP: SHapley Additive exPlanations.

We assessed multicollinearity among input features prior to ML model training, and all VIF values were < 2, indicating no strong multicollinearity (eTable 7). The distributions of SRD derived from the five ML folds are shown in eFigure 1. Across seven ML algorithms to evaluate whether the SRD scores could be predicted from socioeconomic/lifestyle factors in GP1 after controlling age and sex, eXtreme Gradient Boosting (XGBoost) demonstrated the best predictive performance (r: 0.25±0.007; 95% CI: [0.24, 0.25]; R^2^: 0.06±0.003; 95% CI: [0.06, 0.06]; rMSE: 1.08±0.024; 95% CI: [1.08, 1.09]; Figure 3A, eFigure 2). XGBoost was calibrated with a slope of 1.03 and an intercept close to 0 (< 0.001, eFigure 3). Feature importance from SHAP analyses revealed that lower social connection, indexed by less frequent confiding in others, was the main predictor of higher SRD scores. This was followed by physical inactivity factors, including longer hours of sedentary television viewing and fewer days of vigorous physical activity (lasting ≥ 10 minutes/week), and by smoking-related exposure, particularly greater passive smoking outside the home (Figure 3B).

Stratified analyses by age and sex demonstrated that passive smoking exposure was more strongly related to SRD in younger adults (< 65 years) and males, whereas vigorous physical activity was critical among older adults (≥ 65 years) and females (eFigure 4 and 5). Feature contribution rankings were largely stable across age and sex subgroups, though FDR-corrected analyses revealed subgroup-specific differences in contribution weights (eTable 8 and 9). The predictive model outperformed the demographic model (eTable 10), suggesting that the socioeconomic/lifestyle factors provided predictive information beyond demographic characteristics alone. Model performances were comparable with and without linear confound adjustment, indicating minimal risk of data leakage (eTable 11).

### Out-of-sample validation in other independent subsamples

Generalizability was evaluated by applying the GP1-trained models to independent datasets. In both GP2 subsamples, the rCCA model trained in GP1 was applied directly to derive the SRD scores, which were subsequently used as the prediction targets. However, in CD, stronger multivariate associations between SH and depression were observed (canonical correlation *r*_*mean*_ = 0.46 [SD, 0.004]; eFigure 6), indicating a different structure of the SRD phenotype in clinical patients. To avoid underestimating SRD severity in CD and to ensure a representative prediction target, rCCA was retrained within CD using the same framework as GP1, prior to applying the GP1-trained XGBoost model.

**Figure 4:**
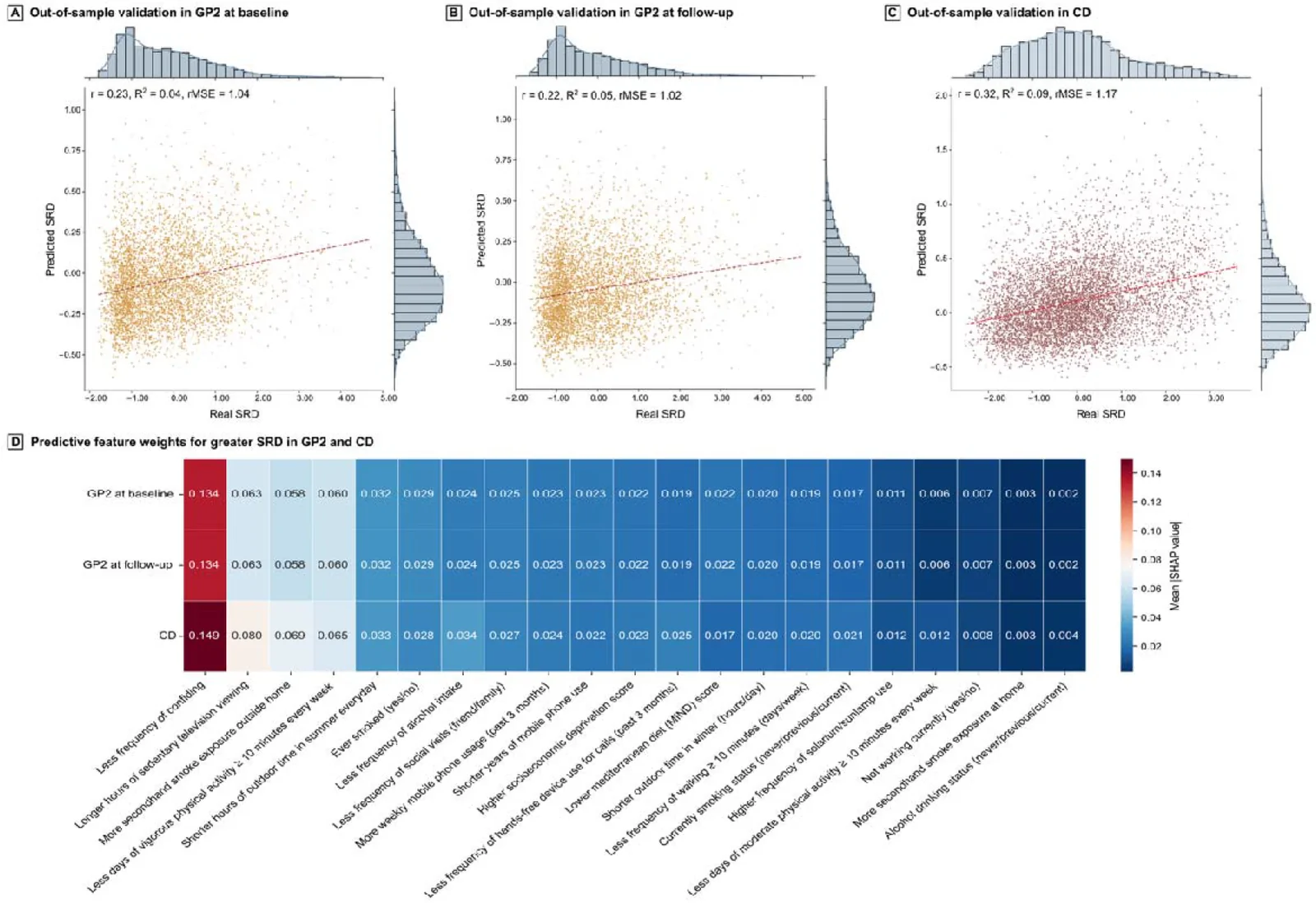
Out-of-sample validation of the prediction model. **A and B.** Out-of-sample validation performances in the independent subsamples (GP2) at baseline and follow-up. **C.** Shows out-ofsample validation performance in the CD. Each point represents an individual participant. The bar plots reflect the distribution of real and predicted SRD scores. Model performance is illustrated using the correlation coefficient (r), coefficient of determination (R^2^), and root mean square error (rMSE). **D.** Weights for each feature in predicting greater SRD in the GP2 at baseline, follow-up, and CD. The colors represent the mean SHAP values for each predictor in the corresponding subsamples. GP: general population subsamples; CD: clinical depression subsamples; SRD: sleep-related depression.

Predictive performance was comparable to GP1 in GP2 at baseline for predicting SRD scores, but lower in GP2 for predicting follow-up SRD scores based on baseline features, with better predictive performance observed in CD (eTable 12, Figure 4A). Across all validation subsamples, the top predictors identified in GP1 remained largely stable. (Figure 4B). The proportion of SH explained by SRD in CD was reported in eTable 6.

### Complementary analyses

**Figure 5.**
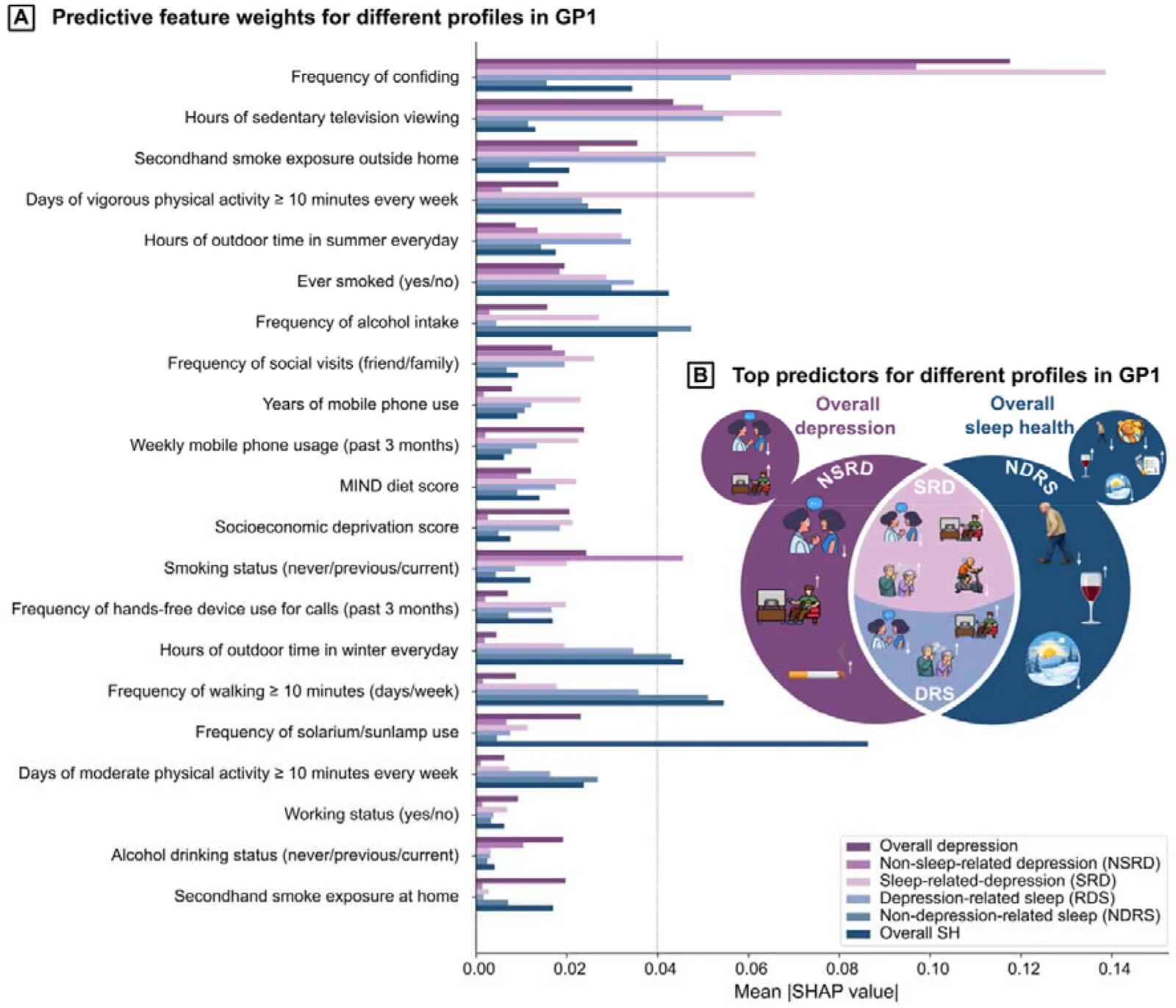
Predictive feature weights for six profiles in GP1. **A.** Feature weights for each sociodemographic /lifestyle factor in predicting different profiles of SH and depression in GP1. Bar length represents the mean absolute SHAP values across the split test sets, reflecting the relative importance of each sociodemographic /lifestyle factor in profile prediction. Colors indicate the different sleep and depression profiles. **B.** This schematic figure illustrates the most important predictors associated with greater symptom severity across six profiles: sleep-related depression (SRD), depression-related sleep (DRS), non-sleep-related depression (NSRD), non-depression-related sleep (NDRS), overall depression (the main dimension of variation in depression), and overall SH (the main dimension of variation in SH). Purple circles represent depression-related profiles, whereas dark blue circles denote sleep-related profiles. *SH: sleep health*.

To further understand different latent profiles of SH and depression, lower predictive performance was observed in DRS, NDRS, and NSRD, whereas higher performance was identified in overall SH and overall depression (eTable 13). Similar to SRD, less frequency of confiding and greater sedentary television viewing merged as the primary predictors of *depression-related profiles* (i.e., NSRD and overall depression), whereas more alcohol consumption frequency, less walking frequency, and less time spent outdoors in winter were the main predictors for *poor sleep-related profiles* (i.e., NDRS and overall sleep disturbances). In contrast, more frequent use of artificial light (sunlamps) was a stronger predictor of sleep disturbances (Figure 5A). These results indicate shared and distinct contributions of socioeconomic/lifestyle features in predicting SH and depression profiles (Figure 5B).

An identical canonical association between SH and depression was observed in non-recurrent depression (eTable 14, eFigure 7), whereas a weaker association was identified in untreated depression (eTable 14, eFigure 8). The predictive performance was comparable, and the top predictors remained stable when excluding recurrent depression, and excluding recurrent depression and antidepressants (eTable 15, eFigures 9–10).

## Discussion

In this large-scale study, we employed a novel combined rCCA and ML approach within a nested cross-validation framework to avoid data leakage while maximizing data utilization. Our aim was to examine whether socioeconomic/lifestyle factors can predict the latent phenotype underlying the interplay between SH and depression. Our predictive models demonstrated moderate but generalizable patterns across cross-sectional and longitudinal GP and CD subsamples. Our rCCA findings identified insomnia and difficulty getting up in the morning as the strongest components linked to depression, such as tiredness and lethargy, in both GP and CD. Despite their robust associations, routine psychotherapies often target either insomnia^46,47^ or depression^1^ in isolation and fail to alleviate the other condition to achieve full remission, suggesting the importance of simultaneously addressing both conditions. Insomnia is a common residual symptom of depression, which may lead to relapses of depression, suicide, and poor prognosis,^48,49^ underscoring the importance of identifying shared influencing factors that may concurrently affect both SH and depression and have investable impacts on treatment outcomes.

Although the protective roles of healthy socioeconomic/lifestyle factors such as physical activity, Mediterranean diet, and social support, and the adverse effects of smoking and alcohol on SH and depression are well established,^21,50–53^ our results further highlighted their role in predicting SRD. Lower frequency of confiding, greater hours of sedentary television viewing, passive smoking exposure outside the home, and fewer days of vigorous physical activity emerged as the top predictors in the present study. Consistently, previous studies found that less confiding frequency, less vigorous physical activity (e.g., swimming or cycling), slower walking pace, and longer sedentary television viewing were associated with more depression,^54,55^ and confiding frequency together with sedentary television viewing were even suggested as causal factors to depression.^55^ Notably, baseline measures of socioeconomic/lifestyle factors moderately predicted subsequent SRD scores assessed approximately 9 years later, underscoring their long-term impacts. The predictive model showed stronger performance in CD, regardless of the effects of recurrent episodes or medication use, suggesting a tighter association between socioeconomic/lifestyle factors and SRD in MDD. This finding was consistent with a meta-analysis reporting larger effect sizes for lifestyle interventions, including physical activity and dietary changes, in MDD patients compared to GP.^56^

Although the top predictors identified in the pooled sample remained stable in age and sex stratified analyses, smoking was more prominent in males and younger adults, while vigorous physical activities were more important in females and older adults, suggesting a non-negligible role of age and sex in modulating the associations between socioeconomic/lifestyle factors and SRD. Prior studies show that SH and depression profiles drastically vary by age and sex,^57,58^ such as physical activities and social support.^59–61^

Socioeconomic and lifestyle factors were more strongly predictive of depression-related profiles than of SH-related profiles. Less frequency of confiding and more sedentary television viewing contributed more strongly to different *depression-related profiles*, whereas less walking frequency and time outdoors in winter, and more alcohol consumption frequency showed greater links with *poor SH-related profiles*. Smoking history was more strongly related to overall sleep disturbances, secondhand smoke exposure outside the home predicted the shared variance of SH and depression (i.e., SRD and DRS), and current smoking status predicted NSRD. Higher frequency of alcohol consumption was predominantly associated with poor *sleep-related profiles*, whereas frequency of artificial light uses predicted SH. It has been shown that alcohol consumption was significantly related to poor SH, including late chronotype, abnormal duration, snoring, insomnia, and daytime napping.^62^ More walking and sunlight exposure have been suggested to be beneficial for reducing sleep disturbances,^60–64^, but less evidence supports an association between exercise intensity and sleep quality improvement.^65^ The frequency of confiding in others is internally associated with social isolation and may drive the model in predicting depression-related profiles.^66^

### Limitations

These findings should be cautiously interpreted, as the observed effect sizes were modest, accounting for only 6–11% of the variance. The generalizability of our findings based on middle-to-old-aged adults to younger adults and adolescents warrants further investigation. SH was not measured by objective assessment and was not stratified by workdays vs non-workdays, given the different sleep patterns across these periods.^24,67,68^ Moreover, because of the different SH-depression associations in GP and CD, the rCCA model was retrained in CD to derive SRD scores as the target for out-of-sample validation of the predictive model; thus, this is not a fully independent out-of-sample validation in CD. Finally, the current observational design precludes any causal inference.

## Conclusions

Robust multivariate associations between SH and depression were consistently observed across four subsamples, and their shared covariance was moderately predictable from socioeconomic/lifestyle factors. Maladaptive lifestyle factors, including lower frequency of confiding, greater sedentary television viewing, secondhand smoke exposure, and less vigorous physical activity, emerged as consistent predictors of higher SRD scores across populations and time points. Our systematic approach disentangled the divergent and convergent roles of socioeconomic/lifestyle factors on the shared and distinct latent dimensions underlying the SH–depression relationships. The generalizability of predictive findings supports the utility of such modifiable factors as potential targets for preventive strategies aimed at reducing the interrelated burden of poor SH and depression.

## Supplementary Material

Supplement 1

## Data Availability

The data used in this study are derived from the UK Biobank resource were approved by the ethical committee of Heinrich Heine University Düsseldorf (2018-317-RetroDEuA). These data are available under restricted access due to ethical approval requirements, participant consent, and data protection regulations. Access can be obtained by researchers through application to the UK Biobank Access Management System (https://www.ukbiobank.ac.uk/), subject to UK Biobank approval. Raw participant-level data are protected and cannot be publicly shared due to data privacy laws. Processed data that do not contain participant-level information are provided with this paper as Source Data files where applicable. Source data supporting this study are provided with this paper.

## Code availability

The analysis code used in this study is publicly available on GitHub at (https://github.com/wenliu1/Socioeconomic-lifestyle_predicting_sleep_depression_profiles).
