# Supplementary material for: Socioeconomic and lifestyle factors predict the association between sleep health and depression
Source: medRxiv. 2026 Jun 29:2026.06.26.26356679. Preprint. [Version 1] doi: 10.64898/2026.06.26.26356679 (PMC13345445; doi:10.64898/2026.06.26.26356679)
Supplement: Supplementary file 1 [file media-1.docx]

**Socioeconomic and lifestyle factors predict the association between sleep health and depression**

**Supplementary file**

**eMethods 1:** 

**Ethical approval and data handling**

The UKB data collection was approved by the Northwest Multicenter Research Ethics Committee, and the present re-analyses of UKB were approved by the ethical committee of Heinrich Heine University Düsseldorf (2018-317-RetroDEuA). Detailed domains of variables included in the rCCA and predictive models: *i)* seven sleep health (SH) components, namely, sleep duration, chronotype, snoring, insomnia, daytime dozing, daytime napping, and ease of getting up in the morning; *ii)* depression, comprising the Recent Depressive Symptoms scale (RDS-4) and the Neuroticism scale (N-12);^1^ *iii)* socioeconomic factors, including social support, working status, and deprivation; and *iv)* lifestyle, including physical activity, diet, smoking, alcohol consumption, digital use, and sun exposure (eTable 1). The MIND diet was suggested to be negatively associated with depression.^2^ To reduce dimensionality and address multicollinearity among many diet variables in UKB, diet quality was quantified using a 9-unit modified MIND score,^3^ which was used as an input feature to predict sleep-related depression (SRD) scores.

**eMethods 2:**

**Exclusion criteira** 

Pariticants were excluded if they had a history of other neuropsychiatric disorders, traumatic brain injury, brain tumors, neurosurgeries, or antipsychotic medication use. Details listed in eTable 2 and eTable 3

**Medication codes from Data-Coding 4**

Data-Coding 4 lists the codes recording medical treatments used by clinical nurses **(**[**https://biobank.ndph.ox.ac.uk/ukb/coding.cgi?id=4&nl=1**](https://biobank.ndph.ox.ac.uk/ukb/coding.cgi?id=4&nl=1)**).**

Codes for antipsychotics in the UK Biobank (UKB) are as follows:

1140868170, 1140928916, 1141152848, 1140867444, 1140879658, 1140868120, 1141153490, 1140867304, 1141152860, 1140867168, 1141195974, 1140867244, 1140867152, 1140909800, 1140867420, 1140879746, 1141177762, 1140867456, 1140867952, 1140867150, 1141167976, 1140882100, 1140867342, 1140863416, 1141202024, 1140882098, 1140867184, 1140867092, 1140882320, 1140910358, 1140867208, 1140909802, 1140867134, 1140867306, 1140867210, 1140867398, 1140867078, 1140867218, 1141201792, 1141200458, 1140867136, 1140879750, 1140867180, 1140867546, 1140928260, 1140927956.

**Codes for antidepressants in the UKB are as follows:**

1140879616, 1140921600, 1140879540, 1140867878, 1140916282, 1140909806, 1140867888, 1141152732, 1141180212, 1140879634, 1140867876, 1140882236, 1141190158, 1141200564, 1140867726, 1140879620, 1140867818, 1140879630, 1140879628, 1141151946, 1140867948, 1140867624, 1140867756, 1140867884, 1141151978, 1141152736, 1141201834, 1140867690, 1140867640, 1140867920, 1140867850, 1140879544, 1141200570, 1140867934, 1140867758, 1140867914, 1140867820, 1141151982, 1140882244, 1140879556, 1140867852, 1140867860, 1140917460, 1140867938, 1140867856, 1140867922, 1140910820, 1140882312, 1140867944, 1140867784, 1140867812, 1140867668.

**eMethods 3:**

**Details for the analysis framework**

The general population 1 subsample (GP1) was randomly split into five outer folds. In each iteration, one outer fold (20%) was used as the CCA training, validation, and test sets for rCCA, while the remaining four folds (80%) formed the ML fold including training, validation, and test sets for predictive models (Figure 1C). Within each CCA fold, data were further split into a training subset (80%) for hyperparameter tuning and a validation subset (20%) for model selection. In parallel, predictive modeling within each ML fold employed a nested cross-validation scheme^4^:  We used 80% of the data as an inner training set, and 20% as the outer test set. The inner training set was further subdivided for hyperparameter tuning (80%) and validation (20%). Predictive model performance was evaluated by averaging root mean square error (rMSE) for all ML test sets across all algorithms, and the best-performing algorithm (lowest rMSE) was selected for subsequent analyses. Training and evaluation within ML folds were repeated twice for stability. To prevent data leakage, all preprocessing steps were performed exclusively withing the training set. Age and sex were regressed out by fitting linear regression models in the training set and the resulting regression parameters were applied to the validation and test sets.^5^ Likewise, continuous and ordinal variables were standardized using the mean and standard deviation estimated from the training set, and these parameters were subsequently applied to the validation and test sets. This procedure was implemented consistently for both rCCA and predictive models.

**eMethods 4:**

**Hyperparameter optimization for rCCA**

The optimal hyperparameters for regularized canonical correlation analysis (rCCA) were searched using GridSearchCV ([https://scikit-learn.org/stable/modules/generated/sklearn.model_selection.GridSearchCV.html](https://scikit-learn.org/stable/modules/generated/sklearn.model_selection.GridSearchCV.html)) according to the mean maximized canonical correlation across validation sets.

**eMethods 5:**

**Robustness of the rCCA model in GP1**

To assess whether the observed canonical association exceeded the ones expected by chance, we evaluated the statistical significance of the multivariate association between sleep health (SH) and depression identified by rCCA using permutation testing. Depression data were independently shuffled across participants for 1,000 iterations, thereby disrupting the original association between SH and depression, while preserving the marginal distributions. For each permutation, the rCCA model with optimised hyperparameters was refit using the shuffled data in the CCA training folds, and the corresponding machine-learning (ML) folds were projected onto the refit rCCA model. Canonical correlation coefficients from both the original and permuted rCCA models were recorded. Empirical *P* values were calculated as the proportion of permuted canonical correlation coefficients exceeding the observed correlation. This procedure was repeated across all ML folds, yielding five *P* values, which were subsequently adjusted for multiple comparisons using false discovery rate (FDR) correction and evaluated at a significance threshold of 0.05.

**eMethod 6:**

**Predictive model training and evaluation**

Hyperparameters for predictive models were optimized by the minimal mean error scores across validation sets. Model performance was evaluated by correlations coefficient (r), coefficient of determination (R^2^), and root of mean squared error (rMSE). To estimate 95% confidence inference (CI) for the metrics, the participants were randomly resampled for 1000 bootstrap iterations to recalculate the metrics for the CI. Last, to calibrate the model performance, a linear regression model was performed, in which the real SRD scores were regressed on predicted SRD scores. Calibration slope and intercept were calculated to assess the correspondence between predicted and observed values. 

**eMethods 7:**

**Complementary age- and sex-specific analyses in GP1**

Given known age- and sex-related differences in both SH and depression,^6,7^ we assessed whether feature contributions varied by age and sex in the best-performing predictive model. Thus, standardized SHapley Additive exPlanations (SHAP) values were compared between younger (< 65 years) and older (≥ 65 years) participants, as well as between male and female participants. Group differences in SHAP values were evaluated using t tests, with FDR correction for multiple comparisons (P < 0.05). An age cutoff of 65 years was selected based on the statutory retirement age in the United Kingdom,^8^ as working status was included as a predictor in the model. 

**eMethods 8:**

**Out-of-sample validation analyses**

To assess the generalizability of our predictive model, out-of-sample validation was conducted for both rCCA and predictive models in GP2 at baseline and follow-up, and the clinical depression subsamples (CD).  For GP2, the depression and SH weights derived from the rCCA analysis in GP1 were applied to the corresponding standardized and deconfounded data in GP2 to project participants onto the latent SH and depression dimensions identified in GP1. Associations between projected SH and depression were then evaluated at GP2 baseline and follow-up to assess the reproducibility and stability of the identified SH–depression relationship in an independent general population cohort. Projection of the GP2 depression data onto the latent dimension identified in GP1 generated SRD scores, reflecting the explicit SH-related depression component. These SRD scores were subsequently used as target variables for the predictive model. As the distribution and multivariate association between SH and depression differed between GP1 and clinical samples, the rCCA model was retrained within CD to obtain a sample-specific SRD phenotype. For out-of-sample validation, the best-performing predictive model trained in GP1 was applied to GP2 (predicting follow-up SRD using baseline predictors) and to subjects in CD using CD-specific SRD scores.

**eMethods 9:**

**Complementary analyses**

To examine how the same socioeconomic and lifestyle contribute to the other profiles of sleep health and depression, compelmentary analyses were conducted for the following latent profiles. 

**Depression-related sleep (DRS):** We defined **Depression-related sleep (DRS)** as a new concept describing the specific dimension of SH that strongly covaries with depression at the individual level. The DRS scores represent the first canonical variate of SH, defined as the linear combination that maximizes its correlation with the corresponding canonical variate of depression at the individual level. So DRS was derived from the same rCCA model used to define SRD in GP1 subsample and was subsequently predicted from the same set of socioeconomic/lifestyle factors using the XGBoost model in predicting SRD.

**Non-sleep-related depression (NSRD):** Multivariate generalized linear models and multivariate logistic regression models were conducted to regress out sleep health, together with age and sex as confounders, from depression variables after standardization for ordinal and categorical variables, respectively. Residualized depression measures were subsequently entered into principal component analysis (PCA) with varimax rotation to identify the first latent dimension independent of SH. The first component,  representing the largest proportion of variance across residualized depression, was defined as NSRD and used as the target for the downstream predictive models. 

        To prevent data leakage, all preprocessing and model fitting procedures were performed within a 5-fold nested cross-validation framework. The dataset was first divided into 5 outer folds, with each fold iteratively serving as the test set. The remaining 4 outer folds constituted the training set and were further split into inner training (80%) and validation (20%) sets for hyperparameter tuning. Multivariate generalized linear models, logistic regression models, and PCA were trained in the same training set and subsequently applied to the held-out test set. Residualized depression measures from the test set were projected onto the PCA model to obtain the first principal component score, which served as the proxy for NSRD. Lastly, the best-performing model for SRD was used to predict the first component of the residualized depression in the test set, using the same socioeconomic/lifestyle features and setting as in predicting SRD. Both the predictive model and PCA were integrated into a nested cross-validation framework. Age and sex were regressed out as confounds in both PCA and predictive models.

**Non-depression-related sleep (NDRS):** The same analytical framework was applied to derive depression independent SH as a proxy measure for NDRS, which was subsequently used as the target for downstream predictive modeling.

**Overall SH:** To obtain the comparable latent dimension of the overall SH for predictive modeling, PCA with varimax rotation was performed within a 5-fold nested cross-validation framework. The first component, representing the largest proportion of variance in SH scores, was subsequently used as the target for a predictive model based on socioeconomic/lifestyle factors. The PCA and predictive model were embedded into a nested cross-validation framework as described previously to avoid data leakage. Age and sex were regressed out as confounds in both PCA and predictive models. 

**Overall depression:** The same analytical framework was used to derive the first component of depression as described for overall SH. 

**eFigure 1. Sleep-related depression (SRD) distribution across five folds.** A-E shows the distributions of SRD scores across the five folds split in the canonical correlation analysis. Histograms represent the frequency distribution of SRD scores within each fold. Vertical dashed lines indicate the mean and ± 1SD of SRD scores for each fold. 

**eFigure 2. Performance of different linear and non-linear ML models in predicting sleep-related depression (SRD) in the general population 1.** Model performance is illustrated using the correlation coefficient (r; A) and the coefficient of determination (R²; B). Each point represents the metric value (r, R2) for an algorithm. The blue and orange bars represent linear models and non-linear models, respectively. ****: *P* < 0.0001. 

*SVR_RBF: support vector regression with radial basis function kernel.*

**eFigure 3: Calibration of XGBoost in GP1.** Each point represents an individual-level SRD score averaged across the test sets; model calibration is summarized by the calibration slope and intercept; histograms depict the distributions of predicted and observed SRD scores. The red solid line represents the fitted line, and the dashed line indicates perfect calibration (predicted SRD =  real SRD).

*GP: general population subsample; SRD: sleep-related depression; SHAP: SHapley Additive exPlanations.*

**eFigure 4. Feature importance for predicting sleep-related depression (SRD) across age-specific subgroups.** SHAP values indicate feature importance in the prediction models in younger (< 65 years; A) and older (≥ 65 years; B) participants in GP1. The x-axis denotes the direction and magnitude of the feature contribution to SRD prediction, where positive values reflect higher predicted SRD and negative values reflect lower predicted SRD. Color reflects feature values: blue indicates low and red indicates high feature scores.

*GP: general population; SHAP: SHapley Additive exPlanations* 

**eFigure 5. Feature importances for predicting sleep-related depression (SRD) across sex-specific subgroups.** SHAP values indicate feature importance in the prediction models in females (A) and males (B) in GP1. The x-axis denotes the direction and magnitude of the feature contribution to SRD prediction, where positive values reflect higher predicted SRD and negative values reflect lower predicted SRD. Color denotes feature values, with blue indicating low and red indicating high feature scores. 

*GP: general population; SHAP: SHapley Additive exPlanations* 

**eFigure 6: First significant latent dimension and loadings for sleep health and depression in clinical depression (CD). A.** Multivariate associations between canonical variates for sleep health (SH) and canonical variates of depression in CD. Each point represents an individual-level SRD score, where scores averaged across the test sets; model performance is illustrated using the correlation coefficient (r); blue and orange bar plots show the distributions of latent scores for SH and depression, respectively. **B.** Mean loadings across five outer CCA folds. Error bars indicate one standard deviation. Greater disturbances in SH loadings were associated with higher depression loadings. Blue represents SH, and orange reflects depression. 

*N-12: neuroticism-12; RDS-4: recent depressive symptoms, indicating depressive symptoms in the past two weeks; SRD: sleep-related depression.*

**eFigure 7:** **First significant latent dimension and loadings for sleep health and depression in clinical depression without recurrent depression. A.** Multivariate associations between canonical variates for sleep health (SH) and canonical variates of depression in non-recurrent depression. Each point represents an individual-level SRD score, where scores averaged across the test sets; model performance is illustrated using the correlation coefficient (r); blue and orange bar plots show the distributions of latent scores for SH and depression, respectively. **B.** Mean loadings across five outer CCA folds. Error bars indicate one standard deviation. Greater disturbances in SH loadings were associated with higher depression loadings. Blue represents SH, and orange reflects depression.

*N-12: neuroticism-12; RDS-4: recent depressive symptoms, indicating depressive symptoms in the past two weeks; SRD: sleep-related depression.*

**eFigure 8: First significant latent dimension and loadings for sleep health and depression in untreated clinical depression. A.** Multivariate associations between canonical variates for sleep health (SH) and canonical variates of depression in individuals with untreated clinical depression. Each point represents an individual-level SRD score, where scores are averaged across the test sets; model performance is illustrated using the correlation coefficient (r); blue and orange bar plots show the distributions of latent scores for SH and depression, respectively. **B.** Mean loadings across five outer CCA folds. Error bars indicate one standard deviation. Greater disturbances in SH loadings were associated with higher depression loadings. Blue represents SH, and orange reflects depression. 

*N-12: neuroticism-12; RDS-4: recent depressive symptoms, indicating depressive symptoms in the past two weeks; SRD: sleep-related depression*

**eFigure 9: Predictive model performance in non-recurrent depression**. **A.** Each point represents an individual; bar plots represent the distribution of real and predicted SRD scores. Model performance is illustrated using the correlation coefficient (r), coefficient of determination (R²), and root mean square error (rMSE). **B.** Weights for each lifestyle and sociodemographic feature in predicting higher SRD in GP1. The length of each bar represents the mean absolute SHAP values across the split test sets, reflecting the importance of each lifestyle and sociodemographic factor in predicting higher SRD. 

*SRD: sleep-related depressive symptoms; SHAP: SHapley Additive exPlanations.*

**eFigure 10: Predictive model performance in untreated depression**. **A.** Each point represents an individual; bar plots represent the distribution of real and predicted SRD scores. Model performance is illustrated using the correlation coefficient (r), coefficient of determination (R²), and root mean square error (rMSE). **B.** Weights for each lifestyle and sociodemographic feature in predicting higher SRD in GP1. The length of each bar represents the mean absolute SHAP values across the split test sets, reflecting the importance of each lifestyle and sociodemographic factor in predicting higher SRD. 

*SRD: sleep-related depression; SHAP: SHapley Additive exPlanations.*

**eTable 1**

| **Supplementary Table 1. Detailed variables for socioeconomic, sleep health, lifestyle, and depression** |  |
| --- | --- |
| **Catergory** | **Name in UKB** |
| Socioeconomics (deprivation) | 189 Townsend deprivation index at recruitment |
| Socioeconomics (social support) | 1031 Frequency of friend/family visits |
|  | 2110 Able to confide |
| Socioeconomics (working status) | 6142 Current employment status |
| Sleep health | 1160 Sleep duration |
|  | 1170 Getting up in the morning |
|  | 1180 Morning/evening person (chronotype) |
|  | 1190 Nap during the day |
|  | 1200 Sleeplessness/insomnia |
|  | 1210 Snoring |
|  | 1220 Daytime dozing/sleeping |
| Depressive symptoms (RDS-4) | 2050 Frequency of depressed mood in the last 2 weeks |
|  | 2060 Frequency of unenthusiasm/disinterest in the last 2 weeks |
|  | 2070 Frequency of tenseness/restlessness in the last 2 weeks |
|  | 2080 Frequency of tiredness/lethargy in the last 2 weeks |
| Depressive symptoms (N-12) | 1920 Mood swings |
|  | 1930 Miserableness |
|  | 1940 Irritability |
|  | 1950 Sensitivity/hurt feelings |
|  | 1960 Fed-up feelings |
|  | 1970 Nervous feelings |
|  | 1980 Worrier/anxious feelings |
|  | 1990 Tense/“highly strung” |
|  | 2000 Worry too long after embarrassment |
|  | 2010 Suffer from “nerves” |
|  | 2020 Loneliness, isolation |
|  | 2030 Guilty feelings |
| Lifestyle (diet) | 1289 Cooked vegetable intake |
|  | 1299 Salad / raw vegetable intake |
|  | 1309 Fresh fruit intake |
|  | 1319 Dried fruit intake |
|  | 1329 Oily fish intake |
|  | 1339 Non-oily fish intake |
|  | 1349 Processed meat intake |
|  | 1359 Poultry intake |
|  | 1369 Beef intake |
|  | 1379 Lamb/mutton intake |
|  | 1389 Pork intake |
|  | 1408 Cheese intake |
|  | 1428 Spread type |
|  | 1438 Bread intake |
|  | 1448 Bread type |
|  | 1458 Cereal intake |
|  | 1468 Cereal type |
|  | 1478 Salt added to food |
|  | 1488 Tea intake |
|  | 1498 Coffee intake |
|  | 1548 Variation in diet |
|  | 2654 Non-butter spread type details |
| Lifestyle (alcohol intake) | 20117 Alcohol drinker status |
|  | 1558 Alcohol intake frequency |
| Lifestyle (smoking) | 20160 Ever smoked |
|  | 20116 Smoking status |
|  | 1269 Exposure to tobacco smoke at home |
|  | 1279 Exposure to tobacco smoke outside the home |
| Lifestyle (physical activity) | 884 Number of days/week of moderate physical activity 10+ minutes |
|  | 904 Number of days/week of vigorous physical activity 10+ minutes |
|  | 864 Number of days/week walked 10+ minutes |
|  | 1070 Time spent watching television (TV) |
| Lifestyle (digital use) | 1110 Length of mobile phone use |
|  | 1120 Weekly usage of mobile phone in the last 3 months |
|  | 1130 Hands-free device/speakerphone use with mobile phone in the last 3 months |
| Lifestyle (sun exposure) | 1050 Time spent outdoors in summer |
|  | 1060 Time spent outdoors in winter |
|  | 2277 Frequency of solarium/sunlamp use |

**eTable 2**

| **Supplementary Table 2: Exclusion criteria** |  |
| --- | --- |
| **Catergory** | **Name in UKB** |
| ICD-10 | A80 Acute poliomyelitis |
|  | A81 Atypical virus infections of the central nervous system |
|  | A83 Mosquito-borne viral encephalitis |
|  | A84 Tick-borne viral encephalitis |
|  | A85 Other viral encephalitis, not elsewhere classified |
|  | A86 Unspecified viral encephalitis |
|  | A87 Viral meningitis |
|  | A88 Other viral infections of the central nervous system, not elsewhere classified |
|  | A89 Unspecified viral infection of the central nervous system |
|  | F00 Dementia in Alzheimer's disease |
|  | F01 Vascular dementia |
|  | F02 Dementia in other diseases classified elsewhere |
|  | F03 Unspecified dementia |
|  | F04 Organic amnesic syndrome, not induced by alcohol and other psychoactive substances |
|  | F05 Delirium, not induced by alcohol and other psychoactive substances |
|  | F06 Other mental disorders due to brain damage and dysfunction, and to physical disease |
|  | F07 Personality and behavioural disorders due to brain disease, damage, and dysfunction |
|  | F09 Unspecified organic or symptomatic mental disorder |
|  | F10 Mental and behavioural disorders due to use of alcohol |
|  | F11 Mental and behavioural disorders due to use of opioids |
|  | F12 Mental and behavioural disorders due to use of cannabinoids |
|  | F13 Mental and behavioural disorders due to use of sedatives or hypnotics |
|  | F14 Mental and behavioural disorders due to use of cocaine |
|  | F15 Mental and behavioural disorders due to use of other stimulants, including caffeine |
|  | F16 Mental and behavioural disorders due to use of hallucinogens |
|  | F17 Mental and behavioural disorders due to use of tobacco |
|  | F18 Mental and behavioural disorders due to use of volatile solvents |
|  | F19 Mental and behavioural disorders due to multiple drug use and use of other psychoactive substances |
|  | F20 Schizophrenia |
|  | F21 Schizotypal disorder |
|  | F22 Persistent delusional disorders |
|  | F23 Acute and transient psychotic disorders |
|  | F24 Induced delusional disorder |
|  | F25 Schizoaffective disorders |
|  | F28 Other nonorganic psychotic disorders |
|  | F29 Unspecified nonorganic psychosis |
|  | F30 Manic episode |
|  | F31 Bipolar affective disorder |
|  | G00 Bacterial meningitis, not elsewhere classified |
|  | G02 Meningitis in other infectious and parasitic diseases classified elsewhere |
|  | G03 Meningitis due to other and unspecified causes |
|  | G04 Encephalitis, myelitis, and encephalomyelitis |
|  | G05 Encephalitis, myelitis, and encephalomyelitis in diseases classified elsewhere |
|  | G30 Alzheimer's disease |
|  | G31 Other degenerative diseases of the nervous system, not elsewhere classified |
|  | G32 other degenerative disorders of the nervous system in diseases classified elsewhere |
|  | G40 Epilepsy |
|  | G41 Status epilepticus |
|  | G46 Vascular syndromes of the brain in cerebrovascular diseases |
|  | G80 Infantile cerebral palsy |
|  | G81 Hemiplegia |
|  | G82 Paraplegia and tetraplegia |
|  | G83 Other paralytic syndromes |
|  | S01 Open wound of the head |
|  | S04 Injury of cranial nerves |
|  | S06 Intracranial injury |
|  | S07 Crushing injury of the head |
|  | S08 Traumatic amputation of part of the head |
|  | S09 Other and unspecified injuries of the head |
|  | F70 Mild mental retardation |
|  | F71 Moderate mental retardation |
|  | F72 Severe mental retardation |
|  | F78 Other mental retardation |
|  | F79 Unspecified mental retardation |
| Self-reported from Data-Coding 6 | 1031 meningeal cancer / malignant meningioma |
|  | 1032 brain cancer / primary malignant brain tumor |
|  | 1081 stroke |
|  | 1082 transient ischaemic attack (TIA) |
|  | 1083 subdural hemorrhage/hematoma |
|  | 1086 subarachnoid haemorrhage |
|  | 1123 sleep apnoea |
|  | 1240 neurological injury/trauma |
|  | 1244 infection of the nervous system |
|  | 1245 brain abscess / intracranial abscess |
|  | 1246 encephalitis |
|  | 1247 meningitis |
|  | 1258 chronic/degenerative neurological problem |
|  | 1259 motor neuron disease |
|  | 1261 multiple sclerosis |
|  | 1262 parkinsons disease |
|  | 1263 dementia/alzheimers/cognitive impairment |
|  | 1264 epilepsy |
|  | 1266 head injury |
|  | 1289 schizophrenia |
|  | 1291 mania/bipolar disorder/manic depression |
|  | 1397 other demyelinating disease (not multiple sclerosis) |
|  | 1409 opioid dependency |
|  | 1410 other substance abuse/dependency |
|  | 1433 cerebral palsy |
|  | 1434 other neurological problems |
|  | 1491 brain hemorrhage |
|  | 1524 spina bifida |
|  | 1583 ischaemic stroke |
|  | 1626 fracture skull/head |
|  | 1659 meningioma / benign meningeal tumor |
|  | 1683 benign neuroma |

**eTable 3**

| **Sample attrition by data category** |  |  |  |  |
| --- | --- | --- | --- | --- |
| Exclusion criterion | GP: N excluded | GP: N remaining | CD: N excluded | CD: N remaining |
| Initial sample after exclusion | - | 386,886 | - | 35,475 |
| Missing demographic variables | 0 | 386,886 | 0 | 35,475 |
| Missing socioeconomic variables | 472 | 386,414 | 57 | 35,418 |
| Missing depression variables | 96,983 | 289,431 | 10,465 | 24,953 |
| Missing sleep health variables | 39,341 | 250,090 | 3,618 | 21,335 |
| Missing working status variables | 1,326 | 248,764 | 132 | 21,203 |
| Missing social support variables | 4,343 | 244,421 | 277 | 20,926 |
| Missing lifestyle variables | 151,829 | 92,592 | 13,472 | 7,454 |
| *Abbreviation: GP: general population; CD: clinical depression; N: number* |  |  |  |  |

**eTable 4**

| **Clinical subsamples for sensitivity analysis** |  |  |  |  |
| --- | --- | --- | --- | --- |
| **Subsamples** | **Sample** | **Age** **mean [SE]** | **Sex (females%)** | **Definition** |
| Non-recurrent depression | 7,363 | 56.04 [7.82] | 69.4% | Recurrent depression was excluded from the clinical depression group, regardless of antidepressant use. |
| Un-treated depression | 2,153 | 55.86 [8.18] | 65.2% | Both individuals with recurrent depression and those using antidepressants while recruited were excluded. |

**eTable 5**

| **Demographic characteristics and feature distributions across the four data domains** |  |  |  |  |
| --- | --- | --- | --- | --- |
|  | **GP1** **(n = 87,405)** | **GP2 at baseline** **(n = 5,187)** | **GP2 at follow-up** **(n = 5,187)** | **CD (n = 7,454)** |
| **Demographic characteristics** |  |  |  |  |
| Age, mean [SE], y | 55.95 [8.09] | 55.17 [7.40] | 64.36 [7.53] | 56.05 [7.82] |
| Sex, No. (%) |  |  |  |  |
| Females | 51.91% | 42.88% | 42.88% | 30.68% |
| **Features** |  |  |  |  |
| Socioeconomic deprivation score | -1.82 [2.77] | -2.18 [2.54] | - | -1.39 [3.00] |
| Frequency of alcohol intake | 4.0 [4.0, 5.0] | 5.0 [4.0, 5.0] | 5.0 [4.0, 5.0] | 4.0 [3.0, 5.0] |
| Alcohol drinking status (never/previous/current) | 2.92% : 2.23% : 94.85% | 1.54% : 1.47% : 96.99% | 2.18% : 2.47% : 95.35% | 4.28% : 5.54% : 90.18% |
| Ever smoked (yes/no) | 56.45% : 43.55% | 55.5% : 44.5% | 51.4% : 48.51% | 58.24% : 41.76% |
| Secondhand smoke exposure outside home | 0.45 [2.12] | 0.32 [1.36] | 0.21 [1.09] | 0.74 [3.84] |
| Smoking status (never/previous/current) | 59.45% : 38.09% : 2.46% | 61.75% : 36.36% : 1.89% | 64.03% : 35.13% : 0.85% | 55.98% : 41.67% : 2.35% |
| Secondhand smoke exposure at home | 0.42 [3.81] | 0.25 [2.75] | 0.12 [1.82] | 0.70 [5.30] |
| MIND diet score | 6.91 [1.07] | 6.98 [1.03] | 6.80 [0.99] | 6.89 [1.13] |
| Frequency of walking ≥ 10 minutes (days/week) | 5.58 [1.82] | 5.57 [1.79] | 6.03 [1.48] | 5.45 [1.98] |
| Days of moderate physical activity ≥ 10 minutes every week | 3.78 [2.26] | 3.74 [2.18] | 4.33 [2.08] | 3.65 [2.37] |
| Days of vigorous physical activity ≥ 10 minutes/week | 2.05 [1.94] | 2.12 [1.85] | 2.27 [1.89] | 1.71 [1.94] |
| Hours of sedentary television viewing | 2.69 [1.41] | 2.49 [1.29] | 2.91 [1.38] | 3.00 [1.61] |
| Frequency of confiding | 3.78 [1.76] | 3.89 [1.70] | 3.77 [1.73] | 3.59 [1.79] |
| Frequency of social visits (friend/family) | 5.30 [1.06] | 5.27 [1.03] | 5.38 [1.04] | 5.41 [1.12] |
| Weekly mobile phone usage (past 3 months) | 1.0 [1.0, 2.0] | 1.0 [1.0, 2.0] | 1.0 [1.0, 2.0] | 1.0 [1.0, 2.0] |
| Frequency of hands-free device use for calls (past 3 months) | 0.0 [0.0, 0.0] | 0.0 [0.0, 0.0] | 0.0 [0.0, 0.0] | 0.0 [0.0, 0.0] |
| Years of mobile phone use | 3.0 [3.0, 4.0] | 3.0 [3.0, 4.0] | 3.0 [3.0, 4.0] | 3.0 [2.0, 4.0] |
| Hours of outdoor time in winter everyday | 2.16 [1.76] | 2.01 [1.57] | 2.27 [1.52] | 2.07 [1.65] |
| Hours of outdoor time in summer every day | 4.11 [2.29] | 3.92 [2.18] | 4.41 [2.11] | 4.07 [2.19] |
| Frequency of solarium/sunlamp use | 0.57 [5.42] | 0.59 [3.66] | 0.24 [2.66] | 0.86 [5.23] |
| Working status (yes/no) | 66.58% : 33.42% | 72.95% : 27.05% | 72.95% : 27.05% | 57.5% : 42.5% |
| Continuous variables are presented as mean [SD], ordinal variables as median [IQR], and categorical variables as percentage. GP: General population; CD: clinically diagnosed depression; SD: standard deviation; IQR: interquartile range. |  |  |  |  |

**eTable 6**

| **Variance in depression and SH explained by opposing canonical variates across folds** |  |  |  |  |  |  |
| --- | --- | --- | --- | --- | --- | --- |
| **Samples** | **Variables** | **Fold 1** | **Fold 2** | **Fold 3** | **Fold 4** | **Fold 5** |
| GP1 | SH | 4.40% | 4.34% | 5.15% | 4.66% | 3.98% |
|  | Depression | 24.00% | 23.91% | 24.90% | 25.35% | 25.57% |
| CD | SH | 3.29% | 3.16% | 3.20% | 2.94% | 3.45% |
|  | Depression | 23.83% | 24.90% | 23.73% | 23.13% | 24.00% |
| *Abbreviation: SH, sleep health; CD: clinical depression subsamples; GP: general population subsamples* |  |  |  |  |  |  |

**eTable 7**

| **Variance inflation factor (VIF) scores for input features in predictive model** |  |  |  |  |  |
| --- | --- | --- | --- | --- | --- |
| **Features** | **VIF fold 1** | **VIF fold 2** | **VIF fold 3** | **VIF fold 4** | **VIF fold 5** |
| Alcohol drinker status | 1.37 | 1.37 | 1.37 | 1.36 | 1.36 |
| Alcohol intake frequency | 1.43 | 1.43 | 1.43 | 1.42 | 1.42 |
| Ever smoked | 1.96 | 1.96 | 1.97 | 1.96 | 1.97 |
| Exposure to tobacco smoke at home | 1.04 | 1.04 | 1.04 | 1.04 | 1.03 |
| Exposure to tobacco smoke outside home | 1.05 | 1.06 | 1.05 | 1.05 | 1.05 |
| Frequency of solarium/sunlamp use | 1.00 | 1.01 | 1.00 | 1.00 | 1.01 |
| Hands-free device/speakerphone use with mobile phone in last 3 month | 1.18 | 1.18 | 1.18 | 1.17 | 1.18 |
| MIND Diet Score | 1.02 | 1.02 | 1.02 | 1.02 | 1.02 |
| Number of days/week of moderate physical activity 10+ minutes | 1.47 | 1.47 | 1.48 | 1.47 | 1.47 |
| Number of days/week of vigorous physical activity 10+ minutes | 1.35 | 1.36 | 1.36 | 1.36 | 1.35 |
| Number of days/week walked 10+ minutes | 1.17 | 1.17 | 1.17 | 1.17 | 1.17 |
| Smoking status | 1.96 | 1.96 | 1.98 | 1.96 | 1.97 |
| Time spend outdoors in summer | 1.90 | 1.93 | 1.91 | 1.91 | 1.93 |
| Time spent outdoors in winter | 1.90 | 1.92 | 1.91 | 1.92 | 1.94 |
| Time spent watching television (TV) | 1.11 | 1.11 | 1.11 | 1.11 | 1.11 |
| Townsend deprivation index at recruitment | 1.05 | 1.05 | 1.05 | 1.05 | 1.05 |
| Weekly usage of mobile phone in last 3 months | 1.27 | 1.28 | 1.28 | 1.28 | 1.28 |
| Working status | 1.21 | 1.21 | 1.21 | 1.21 | 1.21 |
| Years of mobile phone use | 1.14 | 1.14 | 1.14 | 1.14 | 1.14 |

**eTable 8**

| **Group comparison of sex for SHAP values from XGBoost in GP1** |  |  |  |
| --- | --- | --- | --- |
| **Features** | **t -stat** | **p -value** | **p  FDR** |
| Alcohol  intake  frequency | 19.62 | < 0.001 | < 0.001 |
| Alcohol  drinker  status | -18.68 | < 0.001 | < 0.001 |
| Ever  smoked | 27.15 | < 0.001 | < 0.001 |
| Smoking  status | 28.85 | < 0.001 | < 0.001 |
| Exposure  to  tobacco  smoke  at  home | -27.04 | < 0.001 | < 0.001 |
| Exposure  to  tobacco  smoke  outside  home | 7.21 | < 0.001 | < 0.001 |
| MIND  Diet  Score | 0.84 | 0.403 | 0.403 |
| Number  of  days/week  walked  10+  minutes | 1.02 | 0.306 | 0.338 |
| Number  of  days/week  of  moderate  physical  activity  10+  minutes | 3.47 | 0.001 | 0.001 |
| Number  of  days/week  of  vigorous  physical  activity  10+  minutes | -10.53 | < 0.001 | < 0.001 |
| Time  spent  watching  television  (TV) | 0.93 | 0.350 | 0.368 |
| Hands-free  device/speakerphone  use  with  mobile  phone  in  last  3  month | -14.18 | < 0.001 | < 0.001 |
| Weekly  usage  of  mobile  phone  in  last  3  months | -2.52 | 0.012 | 0.016 |
| Years  of  mobile  phone  use | -14.54 | < 0.001 | < 0.001 |
| Frequency  of  solarium/sunlamp  use | -56.57 | < 0.001 | < 0.001 |
| Time  spend  outdoors  in  summer | -23.67 | < 0.001 | < 0.001 |
| Time  spent  outdoors  in  winter | -22.87 | < 0.001 | < 0.001 |
| Working  status | 8.13 | < 0.001 | < 0.001 |
| Frequency  of  friend/family  visits | -1.31 | 0.191 | 0.236 |
| Able  to  confide | 10.07 | < 0.001 | < 0.001 |
| Townsend  deprivation  index  at  recruitment | 1.12 | 0.264 | 0.308 |
| Abbreviation: GP, general population; SHAP: SHapley Additive exPlanations; XGBoost: extreme gradient boosting |  |  |  |

**eTable 9**

| **Group comparison of age for SHAP values from XGBoost in GP 1** |  |  |  |
| --- | --- | --- | --- |
| **Variable** | **t stat** | **p value** | **p fdr** |
| Alcohol intake frequency | 15.60 | < 0.001 | < 0.001 |
| Alcohol drinker status | -3.45 | 0.001 | 0.001 |
| Ever smoked | -16.29 | < 0.001 | < 0.001 |
| Smoking status | -15.06 | < 0.001 | < 0.001 |
| Exposure to tobacco smoke at home | 9.93 | < 0.001 | < 0.001 |
| Exposure to tobacco smoke outside home | 10.35 | < 0.001 | < 0.001 |
| MIND Diet Score | 7.57 | < 0.001 | < 0.001 |
| Number of days/week walked 10+ minutes | -2.87 | 0.004 | 0.005 |
| Number of days/week of moderate physical activity 10+ minutes | 12.48 | < 0.001 | < 0.001 |
| Number of days/week of vigorous physical activity 10+ minutes | -7.89 | < 0.001 | < 0.001 |
| Time spent watching television (TV) | 1.69 | 0.090 | 0.090 |
| Hands-free device/speakerphone use with mobile phone in last 3 month | -11.00 | < 0.001 | < 0.001 |
| Weekly usage of mobile phone in last 3 months | -7.30 | < 0.001 | < 0.001 |
| Years of mobile phone use | -1.89 | 0.059 | 0.062 |
| Frequency of solarium/sunlamp use | 106.98 | < 0.001 | < 0.001 |
| Time spend outdoors in summer | -12.43 | < 0.001 | < 0.001 |
| Time spent outdoors in winter | 3.92 | < 0.001 | < 0.001 |
| Working status | -36.58 | < 0.001 | < 0.001 |
| Frequency of friend/family visits | -3.11 | 0.002 | 0.002 |
| Able to confide | -2.89 | 0.004 | 0.005 |
| Townsend deprivation index at recruitment | 2.34 | 0.020 | 0.022 |
| Abbreviation: GP, general population; SHAP: SHapley Additive exPlanations; XGBoost: extreme gradient boosting |  |  |  |

**eTable 10**

| **Comparison of the full model and demographic model in predicting SRD** |  |  |  |
| --- | --- | --- | --- |
| **Model** | **r (mean±SD)** | **R**^**2**^ **(mean±SD)** | **rMSE (mean±SD)** |
| Full model | 0.25±0.007 | 0.06±0.003 | 1.08±0.024 |
| Demographic model | 0.07±0.009 | 0.00±0.001 | 1.10±0.038 |
| ∆ (Full - Demographic) | 0.18±0.011 | 0.06±0.004 | -0.02±0.042 |
| Full model used socioeconomic and lifestyle factors as features; Demographic model used age, sex, and years of education as features. *SRD: sleep-related depression*. |  |  |  |

**eTable 11**

| **Comparison of predictive performance with and without confounds in predicting SRD** |  |  |  |
| --- | --- | --- | --- |
| **Model** | **r (mean±SD)** | **R**^**2**^ **(mean±SD)** | **rMSE (mean±SD)** |
| Full model with confounds | 0.25±0.007 | 0.06±0.003 | 1.08±0.024 |
| Full model without confounds | 0.25±0.009 | 0.06±0.004 | 1.07±0.036 |
| Both model used the same socioeconomic and lifestyle factors as features. Full model with confounds model controlled age and sex as confounds, whereas full model without confounds model didn't control the prior confounds. *SRD: sleep-related depression.* |  |  |  |

**eTable 12**

| **Model performance in out-of-sample validation** |  |  |  |
| --- | --- | --- | --- |
| **Subsamples** | **r (mean±SD) [95% CI]** | **R**^**2**^ **(mean±SD) [95% CI]** | **rMSE (mean±SD) [95% CI]** |
| GP2 baseline | 0.23±0.003 [0.20, 0.26] | 0.04±0.001 [0.02, 0.05] | 1.04±0.018  [1.01, 1.06] |
| GP2 follow-up | 0.22±0.004 [0.16, 0.21] | 0.05±0.002 [0.02, 0.04] | 1.02±0.017 [1.01, 1.05] |
| CD | 0.32±0.007 [0.31, 0.35] | 0.09±0.004 [0.08, 0.11] | 1.17±0.028 [1.15, 1.18] |
| mean: mean scores across test sets; SD: standard deviation across test sets; *GP: general population subsamples; CD: clinical depression subsamples; CI: conference interval*. |  |  |  |

**eTable 13**

| **Model performance in other profiles of sleep and depression** |  |  |  |
| --- | --- | --- | --- |
| **Profiles** | **r (mean±SD) [95% CI]** | **R**^**2**^ **(mean±SD) [95% CI]** | **rMSE (mean±SD) [95% CI]** |
| DRS | 0.18±0.006 [0.18, 0.19] | 0.03±0.002 [0.03, 0.04] | 1.03±0.007 [1.03, 1.04] |
| NDRS | 0.16±0.007 [0.16, 0.17] | 0.03±0.002 [0.02, 0.03] | 0.99±0.009 [0.98, 0.99] |
| NSRD | 0.17±0.012 [0.17, 0.18] | 0.03±0.004 [0.03, 0.03] | 0.98±0.007 [0.98, 0.99] |
| Overall SH | 0.25±0.006 [0.25, 0.26] | 0.06±0.003 [0.06, 0.07] | 0.97±0.007 [0.96, 0.97] |
| Overall depression | 0.27±0.026 [0.31, 0.33] | 0.07±0.014 [0.10, 0.11] | 0.96±0.014 [0.70, 0.71] |
| mean: mean scores across test sets; SD: standard deviation across test sets; *DRS: depression-related sleep, NDRS: non-depression-related sleep, NSRD: non-sleep-related depression, SH: sleep health; CI: conference interval.* |  |  |  |

**eTable 14**

| **RCCA model performance in CD** |  |
| --- | --- |
| **Subsamples** | **r (mean±SD)** |
| Non-recurrent depression | 0.46±0.007 |
| Untreated depression | 0.44±0.014 |
| mean: mean scores across test sets; *CD: clinical depression subsamples; rCCA: regularized canonical correlation analysis* |  |

**eTable 15**

| **Predictive model performance in CD** |  |  |  |
| --- | --- | --- | --- |
| Subsamples | r (mean±SD) [95% CI] | R^2^ (mean±SD) [95% CI] | rMSE (mean±SD) [95% CI] |
| Non-recurrent depression (prediction) | 0.32±0.012 [0.31, 0.35] | 0.09±0.014 [0.08, 0.11] | 1.19±0.092 [1.16, 1.19] |
| Untreated depression (prediction) | 0.33±0.021 [0.30, 0.38] | 0.09±0.014 [0.08, 0.13] | 1.14±0.198 [1.07, 1.14] |
| mean: mean scores across test sets; *CD: clinical depression subsamples.* |  |  |  |

**Reference:**

1.	Dutt RK, Hannon K, Easley TO, Griffis JC, Zhang W, Bijsterbosch JD. Mental health in the UK Biobank: A roadmap to self-report measures and neuroimaging correlates. *Hum Brain Mapp*. 2022;43(2):816-832. doi:10.1002/hbm.25690

2.	Cherian L, Wang Y, Holland T, Agarwal P, Aggarwal N, Morris MC. DASH and Mediterranean-Dash Intervention for Neurodegenerative Delay (MIND) Diets Are Associated With Fewer Depressive Symptoms Over Time. *J Gerontol A Biol Sci Med Sci*. 2021;76(1):151-156. doi:10.1093/gerona/glaa044

3.	Morris MC, Tangney CC, Wang Y, et al. MIND diet slows cognitive decline with aging. *Alzheimers Dement*. 2015;11(9):1015-1022. doi:10.1016/j.jalz.2015.04.011

4.	Berrar D. Cross-Validation. *Encyclopedia of Bioinformatics and Computational Biology (Second Edition)*. Published online January 1, 2025:638-644. doi:10.1016/B978-0-323-95502-7.00032-4

5.	Komeyer V, Nieto N, Eickhoff SB, Raimondo F, Patil KR. Overview of Challenges in Brain-Based Predictive Modeling: Toward Meaningful Predictive Insights. *Biological Psychiatry*. Published online September 12, 2025. doi:10.1016/j.biopsych.2025.09.003

6.	Epidemiology of sleep health. In: *Sleep and Health*. Academic Press; 2026:13-22. doi:10.1016/B978-0-443-13954-3.00019-X

7.	Luppa M, Sikorski C, Luck T, et al. Age- and gender-specific prevalence of depression in latest-life--systematic review and meta-analysis. *J Affect Disord*. 2012;136(3):212-221. doi:10.1016/j.jad.2010.11.033

8.	Check your State Pension age. GOV.UK. Accessed January 7, 2026. https://www.gov.uk/state-pension-age
